# Comparison of rapid BACpro® II, Sepsityper® kit and in-house preparation methods for direct identification of bacteria from blood cultures by MALDI-TOF MS with and without Sepsityper® module analysis

**DOI:** 10.1007/s10096-019-03654-4

**Published:** 2019-09-07

**Authors:** Munevver Kayin, Berivan Mert, Söhret Aydemir, Volkan Özenci

**Affiliations:** 1 Department of Medical Microbiology, Ege University Medical Faculty of Medicine, Bornova, Turkey; 2 Division of Clinical Microbiology, Department of Laboratory Medicine, Karolinska Institutet, Stockholm, Sweden; 3 Department of Clinical Microbiology F 72, Karolinska Institutet, Karolinska University Hospital, Huddinge, SE 141 86 Stockholm, Sweden

**Keywords:** Blood culture, MALDI-TOF MS, Sepsityper® kit, Rapid BACpro II, Sepsityper® module

## Abstract

There are several approaches available for purifying microorganisms prior to matrix-assisted laser desorption ionization–time of flight mass spectrometry (MALDI-TOF MS) analysis. In the present study, rapid BACpro® II (Nittobo Medical Co., Ltd., Tokyo, Japan), a new application, has been compared with Sepsityper® kit (Bruker Daltonics, Billerica, USA) and an in-house method. Samples were also tested with two modules, standard and Sepsityper®, identified in the Bruker MALDI-TOF MS. The bottles having monomicrobial growth were included in the study according to Gram staining results. In total, two hundred blood culture bottles were included but there was no growth in one of the subcultures so 199 blood culture bottles were studied prospectively. With the standard MALDI-TOF MS analysis, rapid BACpro® II could successfully identify microorganisms in 174/199 (87.4%) of the bottles where Sepsityper® kit and in-house method were successful in 136/199 (68.3%) and 114/199 (57.3%), respectively. When the MALDI-TOF MS data were analysed by Sepsityper® module, the identification rates were increased to 94.4%, 82.1% and 69.8% (*p* < 0.001), respectively. In the Sepsityper® module, 72/73 (98.6%) of Gram-negative and 97/106 (91.5%) of Gram-positive microorganisms were detected by rapid BACpro® II method. The present study shows that rapid BACpro® II is a reliable preparation procedure and has higher rates of identification compared with Sepsityper® kit and in-house method. The use of the Sepsityper® module in blood cultures increases the chance of identification for all three methods studied.

## Introduction

Bloodstream infections (BSIs) remain associated with high morbidity and mortality, even though diagnosis and treatment have greatly improved [1]. Rapid identification of the causative microorganism and its associated resistance pattern is a prerequisite for appropriate antibiotic therapy, which reduces mortality for BSIs. Blood cultures (BCs) are still the gold standard for the diagnosis of BSIs [2]. For patients with BSIs, rapid initiation of the appropriate antimicrobial therapy is essential in reducing mortality and morbidity [3]. Rapid identification of pathogenic microorganisms from blood cultures is crucial for early appropriate antimicrobial therapy [4]. Processing of positive blood cultures is slow due to the subsequent subculture onto solid media for final identification. Although several strategies have been employed to shorten the identification process [5–9], there is an obvious need for reliable, cost-effective and user-friendly methods for rapid identification of microorganisms from blood cultures.

Thus, there is a continuous search for a simple, rapid and cost-effective system for the direct identification of BC pathogens. Many molecular assays have been developed to improve the turnaround time (TAT) of blood cultures using PCR and microarray technologies. Some of these assays are highly sensitive and reduce the TAT significantly and have been used by many clinical laboratories [10]. However, there are important limitations to these assays. The instrument cost is in general high and more importantly the cost per test ranges from approximately 15 to $300 per specimen. In addition, the coverage of microbial species by these assays is limited due to its dependence on the oligo-DNA probes of their panel organisms.

An alternative approach that can overcome these limitations is the use of matrix-assisted laser desorption ionization–time of flight mass spectrometry (MALDI-TOF MS) directly from positive blood cultures [11]. Recently, it was shown that identification of microorganisms directly from positive blood cultures by MALDI-TOF MS significantly reduces time to identification of microorganisms compared with conventional methods [12].

The most important challenge in the use of MALDI-TOF MS for direct identification of bacteria from blood cultures by MALDI-TOF MS is sample preparation. Various approaches have been reported for preparation of the bacteria from the blood culture medium, blood cells and plasma proteins [13, 14]. The vast majority of these methods however are in-house methods that are not standardised and thus are used only locally. There is a need for standardised commercial kits for preparation of bacteria from positive blood cultures.

The Sepsityper® kit (Bruker Daltonics, Billerica, USA) is a commercial pre-treatment kit that selectively destroys blood cells and enriches microorganisms from a positively flagged blood culture. The resulting extracts are suitable for analysis using the MALDI Biotyper system (Bruker Daltonics, Billerica, USA). The Bruker Sepsityper® kit enables identification of the relevant microorganism species from positive bottles within 30 min using the MALDI Biotyper system. Hitherto, published results show that the analytical performance of Bruker Sepsityper® kit is between 76 and 90% [15]. The rapid BACpro II (Nittobo Medical Co., Tokyo, Japan) is a recently introduced pre-treatment kit for effective collection of bacteria with cationic copolymers [13]. The analytical performance of the assay has not been studied in large prospective clinical samples.

Another challenge with using direct MALDI-TOF MS from blood cultures is the analysis of the test results. The scores obtained directly from clinical samples are usually lower than the use of pure cultures from agar plates. The Sepsityper® module is a recently developed system to improve the performance of direct MALDI-TOF MS from blood cultures. Mass spectra are processed in the Sepsityper® software using optimised methods and are reported with adapted score thresholds. The performance of Sepsityper® module in identification of bacteria directly from blood cultures with different preparation protocols has not been studied.

The aim of the present study was to analyse the performance of three different preparation methods; rapid BACpro® II kit, Sepsityper® kit and an in-house method with and without the use of Sepsityper® module for direct identification of bacteria from positive blood culture bottles.

## Materials and methods

The study was performed between April 2018 and June 2018, at Karolinska University Laboratory in Huddinge, Sweden, which serves the southern part of the greater Stockholm area and surrounding cities and suburbs. The laboratory receives blood culture specimens from three tertiary care hospitals: Karolinska University Hospital in Huddinge, Stockholm, South General Hospital, Stockholm, and Södertälje Hospital in Södertälje, with a total of 1569 patient beds. The total number of blood culture bottles processed each year is ca. 90,000. The blood cultures were collected at the clinical wards and then transferred to the laboratory. Three different blood culture bottles, i.e. BacT/Alert FA Plus aerobic, BacT/Alert PF Plus pediatric and BacT/Alert FN Plus anaerobic bottles were used. The BC bottles were incubated in BacT/Alert 3D (bioMérieux, Durham, NC, USA) blood culture systems until they yielded a positive signal or for a final period of 5 days. When the blood culture bottles yielded a positive signal, Gram staining was performed. After Gram staining, only BC bottles with monomicrobial growth were included in the study. The three methods were assessed for blood culture broth processing prior to MALDI-TOF MS analysis.

### Conventional microbiological method

According to the results of the Gram staining, the specimens from the positive BC bottles were subcultured onto the relevant agar plates. The microorganisms grown on the agar plates after overnight culture at 37 *°*C at relevant atmosphere were identified by Bruker MALDI-TOF MS (Bruker Daltonics, Billerica, MA, USA). The conventional method was used as a reference method.

### Sample preparation for direct MALDI-TOF MS identification

**Fig. 1:**
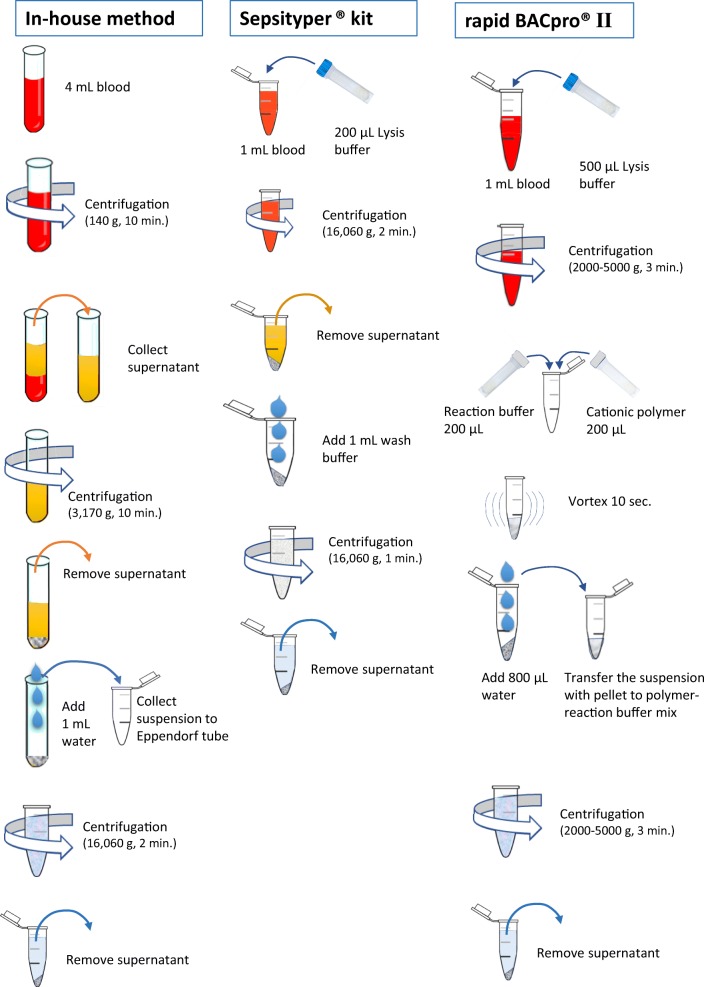
Sample preparation steps for the three methods included in the study

A bacterial pellet that was free from human blood cells and proteins, and culture medium is produced by centrifugation and washing steps using in-house method, Sepsityper® lysis solution in Sepsityper® kit and cationic polymers in rapid BACpro® II kit. Figure 1 depicts the three protocols that were used in the present study.

### In-house method

Four millilitres of the blood culture broth was centrifuged at 140*g* for 10 min. The supernatant was collected in a new tube then centrifuged at 3170*g* for 10 min. The supernatant was removed, and the pellet was washed with 1 mL of sterile water and the suspension was collected in an Eppendorf tube then centrifuged at 16,060*g* for 2 min. The supernatant was removed and discarded.

### Sepsityper® kit

One millilitre of blood culture broth was transferred to an Eppendorf tube and 200 μL lysis buffer was added. The mix was centrifuged for 2 min at 16,060*g*. The supernatant was removed, and 1 mL washing buffer was added, and the pellet was resuspended and was centrifuged for 1 min at 16,060*g*. The supernatant was removed by pipetting**.**

### Rapid BACpro® II

One millilitre of the blood culture broth was taken into the 2-mL tube and 500 μL lysis buffer was added before it was centrifuged at 5000*g* for 3 min and the supernatant was removed as completely as possible. During centrifugation, 200 μL cationic polymer suspension and 200 μL reaction buffer were prepared in the 1.5-mL tube. The pellet was then resuspended in the polymer-reaction buffer mix and centrifuged at 5000*g* for 1 min.

### Protein extraction step

The pellet obtained with the above-described protocols was resuspended in 300 μL HPLC-quality water. Subsequently, for each of the three methods, 900 μL 100% ethanol was added and the suspension was centrifuged at 16,060*g* for 2 min. The supernatant was discarded, and the pellet resuspended in 20–50 μL 70% formic acid and the same amount of 100% acetonitrile (Table 1). After centrifugation at 16,060*g* for 1 min, 1 μL of the supernatant was spotted onto the MALDI target plate (Bruker Daltonics, Billerica, MA, USA) and allowed to dry. Then, 1 μL of matrix solution was overlaid before it was analysed.

**Table 1:** Description of the three protocols for bacterial identification directly from blood cultures included in the study

	Blood culture broth volume	Preparation of bacterial pellet	Protein extraction
In-house	4 mL	1. Centrifugation (10′ for 140*g*) 2. Centrifugation (10′ for 3170*g*) + distilled water (1 mL)	Ethanol (900 μL) + Formic acid (20 μL) + acetonitrile (20 μL)
Sepsityper®	1 mL	1. Centrifugation (2′ for 16,060*g*) + lysis buffer (200 μL) 2. Centrifugation (1′ for 16,060*g*) + wash buffer (1 mL)	Ethanol (900 μL) + Formic acid (20 μL) + acetonitrile (20 μL)
Rapid BACpro® II	1 mL	1. Centrifugation (3′ for 5000*g*) + 500 μL lysis buffer 2. Centrifugation (1′ for 5000*g*) + cationic solution (400 μL) + distilled buffer (800 μL)	Formic acid (30 μL) + acetonitrile (100 μL)

### Analysis with MALDI-TOF MS

Spectra was automatically captured and analysed by the Microflex LT MALDI-TOF system equipped with MALDI Biotyper analysis software and database (version 2.0, IVD MALDI Biotyper database 4613). Three MALDI spots were analysed for each preparation protocol in order to determine the reproducibility of the method. Two different MALDI scoring methods were used for analysis: standard module and Sepsityper® module. Each sample was registered as a “Sepsityper® sample type” enabling the module to a specific data processing considering account the undesired mass spectrum peaks originating from blood cells. The acquired spectra were compared with the spectra from the reference database and an identification result was associated with an identification score used to express the degree of spectral concordance. For standard module and Sepsityper® module, acceptable species identification scores were ≥ 1.70 and ≥ 1.60, respectively. Scores above 2.0 were regarded as reliable identification at species level. Sepsityper samples giving scores below the cut-off values were recorded as having no identification by direct MALDI-TOF MS.

### Statistical analysis

The performance of rapid BACpro® II, Sepsityper® kit and the in-house method was compared by using the chi-square test. Fisher’s exact test was used in comparing identification rates in two different analysis modules. Statistical significance was determined with *p* value < 0.05. The data were analysed using the GraphPad Prism for Windows version 5.04.

## Results

In total, 200 positive blood culture bottles were included in the study, prospectively. One sample was excluded because there was no growth on agar plates after subcultures from the positive blood culture bottle. Identification of microorganisms directly from positive blood cultures by MALDI-TOF MS was performed using three different sample preparation methods studied. There were 123 BacT/ALERT FA Plus, 70 BacT/ALERT FN Plus and 6 BacT/ALERT PED Plus blood culture bottles analysed. In total, 115 (57.8%) Gram-positive and 84 (42.2%) Gram-negative bacteria were detected. All 199 microorganisms were identified by MALDI-TOF MS from agar plates with standard module after overnight cultures and used as a reference method. In comparing the three preparation methods, 199 and 179 samples were analysed with standard module and Sepsityper® module, respectively. Table 2 depicts the individual microorganisms isolated from the positive blood cultures.

**Table 2:** Performance of MALDI-TOF MS identification of 199 monomicrobial blood cultures by three methods compared with definitive identification

	Reference method		Rapid BACpro® II		Sepsityper® kit		In-house method	
Organism	Standard module	Sepsityper® module	Standard module	Sepsityper® module	Standard module	Sepsityper® module	Standard module	Sepsityper® module
*Escherichia coli*	46	41	46	41	45	40	46	41
*Klebsiella pneumoniae*	6	6	6	6	6	6	6	5
*Bacteroides fragilis*	4	4	4	4	0	4	4	4
*Achromobacter xylosoxidans*	2	2	2	2	2	2	2	2
Gram-negative *bacilli*	2		0		0		0	
*Hafnia alvei*	2	2	2	2	2	2	2	2
*Klebsiella oxytoca*	3	3	3	3	2	3	3	3
*Pantoea septica*	2	2	2	2	1	1	1	1
*Proteus mirabilis*	2	2	2	2	1	2	1	1
*Pseudomonas aeruginosa*	2	2	2	2	2	2	2	2
*Stenotrophomonas maltophila*	2	1	2	1	1	1	1	1
*Acinetobacter baumannii*	1	1	1	1	0	0	1	1
*Actinobaculum massiliense*	1		1		0		0	
*Aerococcus sanguinicola*	1		0		0		0	
*Aerococcus urinae*	1	1	1	1	0	1	0	0
*Bacteriodes thetaiotaomicron*	1	1	1	1	0	0	1	1
*Bacteriodes vulgatus*	1		1		0		1	
*Campylobacter coli*	1	1	0	0	0	0	0	0
*Klebsiella variicola*	1	1	1	1	1	1	1	1
*Moraxella osloensis*	1	1	0	1	0	1	0	0
*Odoribacter splanchnicus*	1	1	1	1	0	0	1	1
*Raoultella ornithinolytica*	1	1	1	1	1	1	1	1
Gram-negative *bacteria*	84	73	79	72	64	67	74	67
*Staphylococcus epidermidis*	33	30	29	30	20	23	13	21
*Staphylococcus aureus*	22	19	22	19	21	18	9	8
*Staphylococcus hominis*	6	6	5	5	4	4	1	3
*Streptococcus pneumoniae*	6	6	2	3	1	1	4	4
*Enterococcus faecium*	5	5	5	5	2	5	1	4
*Staphylococcus haemolyticus*	5	5	5	5	4	5	1	1
*Staphylococcus capitis*	3	2	3	2	1	1	1	1
*Streptococcus pyogenes*	3	4	3	4	2	3	1	1
*Micrococcus luteus*	2	2	1	2	1	1	1	1
*Propionibacterium acnes*	2	3	1	2	2	2	1	1
*Streptococcus anginosus*	5	3	3	2	1	2	0	1
*Streptococcus salivarius*	2	2	1	1	0	1	0	0
*Streptococcus sanguinis*	2	1	1	1	1	1	0	0
*Bacillus thuringiensis*	1	1	1	1	0	0	0	0
*Beta haem strep. gr.G*	1		0		0		0	
*Brevibacterium casei*	1	1	1	1	1	1	1	1
*Corynobacterium amycolatum*	1	1	1	1	1	1	0	0
*Corynobacterium mucifaciens*	1	1	1	1	0	0	0	0
*Enterococcus faecalis*	1	1	1	1	1	1	1	1
*Enterococcus gallinarum*	1	1	1	1	1	1	0	0
*Gemella haemolyticus*	1	1	1	1	1	1	0	1
*Parvimonas micra*	1	1	0	1	0	0	0	0
*Rothia mucilaginosa*	1	1	1	1	1	1	0	0
*Staphylococcus simulans*	1	1	1	1	1	1	1	1
*Staphylococcus warneri*	1	1	0	1	1	1	0	1
*Staphylococcus xylosus*	1	1	1	1	1	1	1	1
*Streptococcus agalactiae*	1	1	1	1	1	1	1	1
*Streptococcus bovis*	2	2	1	1	0	1	0	2
*Streptococcus dysgalactiae*	1	1	0	0	1	1	0	1
*Streptococcus mitis*	2	2	2	2	1	1	2	2
Gram-positive *bacteria*	115	106	95	97	72	80	40	58
No identification	0	0	25	10	63	32	85	54
Total	199	179	199	179	199	179	199	179

### Comparison of the three methods by using the standard module

**Fig. 2:**
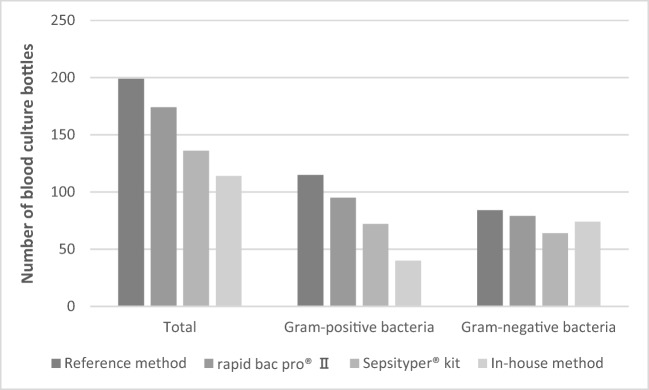
Sample preparation with three different methods for identification of bacteria directly from blood culture bottles by MALDI-TOF MS analysed by standard module

The rapid BACpro® II could correctly identify the bacteria in 174/199 (87.4%) blood culture bottles using the standard module. The Sepsityper® kit and the in-house method could identify bacteria in 136/199 (68.3%) and 114/199 (57.3%) bottles, respectively. There was a significant difference in identification rates among the three methods (*p* < 0.0001). Rapid BACpro® II had significantly higher identification rates than the Sepsityper® kit and the in-house method (*p* < 0.0001 for both comparisons). Sepsityper® kit could detect higher numbers bacteria than in-house method (*p* < 0.05) (Fig. 2, Table 2).

In total, 115 Gram-positive bacteria were analysed by the three methods. The rapid BACpro® II, Sepsityper® kit and in-house method could identify 82.6% (95/115), 62.6% (72/115) and 34.8% (40/115) of the Gram-positive bacteria, respectively (*p* < 0.0001) (Fig. 2). The rapid BACpro® II could identify higher numbers of Gram-positive bacteria than the Sepsityper® kit and in-house method (*p* = 0.001 and *p* < 0.0001, respectively). Sepsityper® kit detected higher numbers of Gram-positive bacteria than in-house method (*p* < 0.0001).

**Fig. 3:**
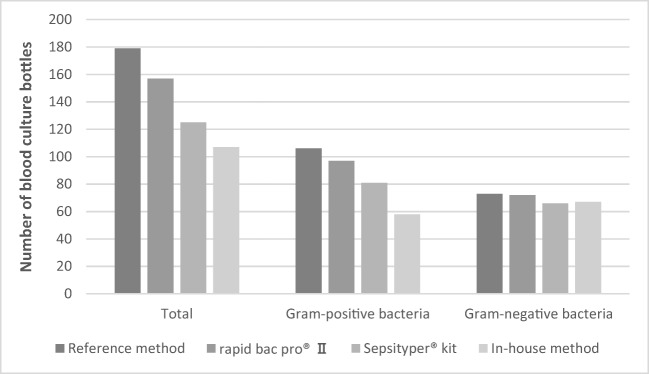
Sample preparation with three different methods for identification of bacteria directly from blood culture bottles by MALDI-TOF MS analysed by Sepsityper® module

Regarding Gram-negative bacteria, 84 samples were analysed. The rapid BACpro® II, Sepsityper® kit and in-house method could identify 94% (79/84), 76.2% (64/84) and 88.1% (74/84) of the isolates, respectively (*p* < 0.005) (Fig. 3). The rapid BACpro® II could identify higher numbers of Gram-negative bacteria than the Sepsityper® kit (*p* < 0.005). No statistically significant difference was found between rapid BACpro® II and the in-house method for Gram-negative bacteria.

### Comparison of the three methods by using the Sepsityper® module

Direct MALDI-TOF MS results from 179 blood culture bottles were also analysed by the Sepsityper® module. The rapid BACpro® II, Sepsityper® kit and in-house method could identify bacteria in 94.4% (169/179), 82.1% (147/179) and 69.8% (125/179) blood cultures, respectively (< 0.0001). Overall, rapid BACpro II could identify higher numbers of bacteria than the Sepsityper® kit and in-house method (*p* < 0.05 and *p* < 0.0001, respectively). Sepsityper® kit detected higher numbers of bacteria than in-house method with Sepsityper® module (*p* < 0.0001) (Fig. 3, Table 2).

Regarding the Gram-positive bacteria, the rapid BACpro® II, Sepsityper® kit and in-house method could identify bacteria in 91.5% (97/106), 75.5% (80/106) and 54.7% (58/106) blood cultures, respectively (*p* < 0.0001) (Fig. 2). The rapid BACpro® II could identify higher numbers of Gram-positive bacteria than the Sepsityper® kit and in-house method (*p* < 0.005 and *p* < 0.0001, respectively). Sepsityper® kit detected higher numbers of bacteria than in-house method with Sepsityper® module (*p* < 0.005) (Fig. 3).

In total, 73 blood culture bottles with Gram-negative bacteria were analysed by Sepsityper® module. The rapid BACpro® II, Sepsityper® kit and in-house method could correctly identify 98.6% (72/73), 91.8% (67/73) and 91.8% (67/73) of the Gram-negative bacteria, respectively (Fig. 3). No statistically significant difference was found among the three methods in identifications of Gram-negative bacteria.

### MALDI Biotyper cut-off values

The three preparation methods were also compared in identification bacteria at species level, i.e., with MALDI-TOF MS scores > .0. The rapid BACpro® II, Sepsityper® kit and in-house method could identify 121/199 (60.8%), 86/199 (43.2%) and 69/199 (34.7%) of the isolates with scores > 2.0, respectively (*p* < 0.0001). The rapid BACpro® II could identify higher numbers of bacteria with scores above 2.0 than the Sepsityper® kit and in-house method (*p* < 0.001 and *p* < 0.0001, respectively). Sepsityper® kit detected higher numbers of bacteria with scores above 2.0 than in-house method (*p* < 0.005) (Table 3).

**Table 3:** The MALDI-TOF MS cut-off scores obtained with the three different separation methods

	Rapid BACpro® II *n* (%)		Sepsityper® kit *n* (%)		In-house method *n* (%)	
Cut-off value	Standard module	Sepsityper® module	Standard module	Sepsityper® module	Standard module	Sepsityper® module
≥ 2.00	121 (60.8)	133 (74.3)	86 (43.2)	102 (57.0)	69 (34.7)	68 (38.0)
1.70–1.99	53 (26.6)	29 (16.2)	50 (25.1)	29 (16.2)	45 (22.6)	48 (26.8)
1.60–1.69	0 (0)	7 (3.9)	0 (0)	16 (8.9)	0 (0)	9 (5.0)
No identification	25 (12.6)	10 (5.6)	63 (31.6)	32 (17.9)	85 (42.7)	54 (30.2)
Total	199 (100)	179 (100)	199 (100)	179 (100)	199 (100)	179 (100)

When the results were analysed by the Sepsityper® module, the rapid BACpro® II could identify 133/179 (74.3%) isolates at species level whereas the Sepsityper® kit and in-house method could identify 102/179 (57.0%) and 68/179 (38.0%) isolates with scores > 2.0 with the Sepsityper® module (*p* < 0.0001) (Table 3). The rapid BACpro® II could identify higher numbers of bacteria with scores above 2.0 than the Sepsityper® kit and in-house method (*p* < 0.001 and *p* < 0.0001, respectively). Sepsityper® kit detected higher numbers of bacteria with scores above 2.0 than in-house method (*p* = 0.0005).

Regarding the minimum cut-off score for whether any ID was obtained, we employed scores above 1.7 and 1.6 for the standard and Sepsityper® modules, respectively (i.e., scores below these values are considered not giving proper ID). With these minima cut-off scores, in both the standard and Sepsityper® modules, processing the samples with rapid BACpro® II gave a smaller number of failed ID (i.e., no ID given) as compared with Sepsityper® kit and the in-house method (Table 3).

### Comparison of the standard module with the Sepsityper® module

The performance of the standard and Sepsityper® modules was compared in 179 blood culture bottles (Gram negative 73, Gram positive 106) that were analysed by both modules. Table 4 depicts the results obtained by the standard module with the Sepsityper® module analysis. Of the 537 analysis performed from all three preparation methods, Sepsityper® module had significantly higher numbers of overall correct identification results than the standard module, 441 (82.1%) vs. 389 (72.4%) (*p* < 0.0005). The rapid BACpro® II and Sepsityper® kit had significantly increased correct identification with Sepsityper® module compared with the standard module, from 87.7 to 94.4% and from 69.8 to 82.1%, respectively (Table 4). There was also a tendency for increased identification rates by in-house method with Sepsityper® module, not reaching statistical significance, from 59.8 to 69.8% (*p* = 0.06).

**Table 4:** Comparison of the standard module with the Sepsityper® module in identification of bacteria directly from blood culture bottles by MALDI-TOF MS

Organism	Rapid BACpro® II *n* (%)		Sepsityper® kit *n* (%)		In-house method *n* (%)	
	Standard module	Sepsityper® module	Standard module	Sepsityper® module	Standard module	Sepsityper® module
Gram-positive bacteria (*n* = 106)	86 (81.1)	97 (91.5)	65 (61.3)	81 (76.4)	39 (36.8)	58 (54.7)
Gram-negative bacteria (*n* = 73)	71 (97.3)	72 (98.6)	60 (82.2)	66 (90.4)	68 (93.2)	67 (91.8)
Total (*n* = 179)	157 (87.7)	169 (94.4)	125 (69.8)	147 (82.1)	107 (59.8)	125 (69.8)

Identification is defined by concordance with the reference method. Correct identification is defined by scores ≥ 1.7 or ≥ 1.6 for the standard or Sepsityper® module, respectively

When Gram-positive and Gram-negative bacteria analysed separately, the improvement was remarkable in Gram-positive bacteria for all three methods. The rapid BACpro® II, Sepsityper® kit and the in-house method could identify significantly higher numbers of Gram-positive bacteria with the Sepsityper® module than the standard module, 86/106 (81.1%) vs. 97/106 (91.5%), 65/106 (61.3%) vs. 81/106 (76.4%) and 39/106 (36.8) vs. 58/106 (54.7%), respectively (*p* < 0.05, *p* < 0.05 and *p* = 0.01, respectively). No statistically significant difference was observed in identification of Gram-negative bacteria between the standard module and Sepsityper® module for all three methods studied.

### The organisms that could not be identified

For all three preparation methods analysed, many of the isolates that could not be identified were Gram-positive bacteria. In total, no identification was observed in 221 tests with Gram-positive and only 48 tests with Gram-negative bacteria. All three methods had low identification rates for streptococci and enterococci. The rapid BACpro® II could not correctly identify 11/31 of streptococci and enterococci with standard module. This rate was 20/31 with the Sepsityper® kit and 22/31 with the in-house method (Table 5). Another group of bacteria with high rates of failed identification was staphylococci and micrococci, especially with the in-house method.

**Table 5:** The organisms that could not be identified with the three methods

Organism	Rapid BACpro® II		Sepsityper® kit		In-house method	
Standard results	Standard module	Sepsityper® module	Standard module	Sepsityper® module	Standard module	Sepsityper® module
Staphylococci	6	1	19	11	45	28
Micrococci	1	0	1	1	1	1
Streptococci	11	7	17	10	17	10
Enterococci	0	0	3	0	5	2
Other Gram-positives	2	1	3	4	7	7
Gram-positive bacteria	20	9	43	26	75	48
*Enterobacteriaceae*	0	0	4	2	2	3
Other Gram-negatives	5	1	16	4	8	3
Gram-negative bacteria	5	1	20	6	10	6
Total	25	10	63	32	85	54

### Turnaround time

The total turnaround time for identification of bacteria from a single blood culture bottle with direct MALDI-TOF MS was approximately 30 min for each of the three preparation methods studied.

## Discussion

Identification of bacteria directly from positive blood cultures is regarded as a significant improvement in microbiological diagnostic of BSIs. However, the rapid methods are in general complementary to standard methods and the high instrument and test kit costs hinder the implementation of these methods in the clinical routine [16, 17]. In the recent years, MALDI-TOF MS has become one of the most important tools in identification of microorganisms in clinical microbiology laboratories. The advantages of the method include extensive coverage, low cost and user-friendliness. The implementation of direct MALDI-TOF MS from blood cultures as a rapid identification method is therefore attractive since the method has already established in the routine, and the instrument cost will therefore not be an additional burden for the laboratories. However, MALDI-TOF MS identification of bacteria is dependent on their unique protein profile and requires in general pure colonies [18]. Therefore, accurate MALDI-TOF MS identification of bacteria from blood culture bottles requires separation of bacteria from other disturbing signals including human cells, culture media and other debris that are present in the blood culture broth [17, 19, 20]. Several previous studies have focused on development in-house methods for sample preparation with varying results. However, none of the methods are widely used since there is no standardisation and the performance of these in-house methods depends on operators’ skills. It is important to analyse the performance of standardised commercial methods in sample preparation.

The present study is the first to perform a three-way comparison of the recently introduced rapid BACpro® II with two different methods for preparing positive blood cultures for direct MALDI-TOF MS analysis with and without the recently introduced Sepsityper® module. The present data shows that the rapid BACpro® II has significantly higher levels of identification of bacteria directly from blood culture bottles with MALDI-TOF MS compared with the Sepsityper® kit and in-house preparation method, 87.4% vs. 68.3% and 57.3%, respectively.

Almost all the previously published studies have showed that the identification of Gram-negative bacteria is more successful than Gram-positive bacteria by direct MALDI-TOF MS from blood cultures [21–23]. Although the underlying reason for this discrepancy is not known, it is reasonable to suggest that the bacterial cell wall components may play a role. In line with several previously published studies, the present study shows that all three separation methods have higher performance in identification of Gram-negative bacteria compared with Gram-positive bacteria [21–23]. Among the bacteria that could not be identified by the three methods analysed, the two groups, staphylococci and streptococci, dominated.

The use of Sepsityper® module in analysis of the direct MALDI-TOF MS results improved the overall identification rates of bacteria from positive blood cultures significantly. The rapid BACpro® II kit, Sepsityper® kit and in-house method could identify bacteria in 94.4%, 82.1% and 69.8% using Sepsityper® module. Interestingly, the increased correct identification rates with the use of Sepsityper® module was solely on Gram-positive bacteria. No difference was observed between the standard module and Sepsityper® in identification of Gram-negative bacteria. The underlying reason for this discrepancy remains to be studied. It is however possible to assume that the poor identification rates in Gram-positive bacteria with standard module might open the possibility to further improvement using Sepsityper® module.

The sample volume reflecting the inoculum size is probably one of the most important factors in the performance of direct MALDI-TOF MS from blood culture methods. Previous studies included a range of sample volumes, from 0.2 [24] to 8 mL [25]. In the present study, we used 4 mL blood culture broth for the in-house method and 1 mL of each for the rapid BACpro® II and the Sepsityper® kit. The present data with higher performances with the rapid BACpro® II and the Sepsityper® kit compared with the in-house method shows that the sample volume of 1 mL is enough if the preparation method is effective.

There are limitations in the present study. The study did not include any blood cultures with polymicrobial growth or yeast. However, the goal of the study was to compare the performances of the methods and the analysis on monomicrobial positive blood cultures with bacteria. Recently, the increased identification of bacteria with polymicrobial growth using the modified Sepsityper® module was reported [26]; second, the clinical impact of the separation methods was not analysed.

The present data were not used in guiding antimicrobial treatment. However, hitherto, published studies have showed that direct MALDI-TOF MS pathogen identification from positive blood culture have a positive impact on antimicrobial treatment in patients with BSIs [21, 22]. It is reasonable to suggest that rapid BACpro® II with the present promising clinical results might be useful in rapid microbiological diagnostic of blood cultures in the clinical routine. Similarly, the Sepsityper® module improved the yield of accurate identification of bacteria from BC bottles in the studied material.

Hitherto, published studies showed variable results in the performance of different preparation methods ranging between 61 and 98% [21]. The analytical performance of the method, the experimental design, and the epidemiology of bacterial spp. causing bloodstream infections are important factors that may influence the outcome of the method tested. Therefore, it is problematic to compare different studies for the performance of different separation methods used in direct identification of bacteria from cultures. However, the present study was performed in a tertiary care hospital prospectively and included more than sixty different bacterial species and included representative material for the comparison of the three methods included. Therefore, it is reasonable to suggest that the present data is a representative.

In conclusion, the present study shows that (i) the rapid BACpro® II kit with a performance of identification of 94.4% of the bacteria directly from blood cultures is a promising approach for the clinical use and (ii) the use of Sepsityper® module improves the performance of direct identification of bacteria by MALDI-TOF MS regardless of the preparation method used. Further studies analysing the impact of these methods in the presence of Sepsityper® module on early treatment and on the outcome of bloodstream infections are warranted.
